# Duchenne’s Muscular Dystrophy: The Role of Induced Pluripotent Stem Cells and Genomic Editing on Muscle Regeneration

**DOI:** 10.7759/cureus.10600

**Published:** 2020-09-22

**Authors:** Vanessa May, Ashley A Arnold, Sukrut Pagad, Manoj R Somagutta, Saijanakan Sridharan, Saruja Nanthakumaran, Bilal Haider Malik

**Affiliations:** 1 Department of Research, California Institute of Behavioral Neurosciences & Psychology, Fairfield, USA; 2 Department of Internal Medicine, Larkin Community Hospital, Hialeah, USA; 3 Department of Internal Medicine, California Institute of Behavioral Neurosciences & Psychology, Fairfield, USA

**Keywords:** duchennes muscular dystrophy, stem cell therapy, induced pluripotent stem cell therapy, muscular dystrophy, genetic editing

## Abstract

There are two types of well-known muscular dystrophies: Duchenne's muscular dystrophy (DMD) and Becker's muscular dystrophy. This article focuses on the X-linked recessive disorder of Duchenne's muscular dystrophy, which primarily affects children at age four, with a shortened life span of up to 40 years. A defective dystrophin protein lacking the gene dystrophin is the primary cause of the disease pathophysiology. This defect causes cardiac and skeletal muscle down-regulation of dystrophin, leading to weak and fibrotic muscles. The disease is currently untreatable, so most kids die due to cardiac failure in their late 30's.

This review presents current treatment options, based on previous studies conducted over the last five years. We used the PubMed database to analyze and review the most important investigations. We also included an analysis of induced pluripotent stem cell therapy vs. genetic therapy using the mdx mouse model. We have discovered promising results on mdx mouse models to date and excited about the potential for where further clinical human trials can go.

## Introduction and background

Duchenne's muscular dystrophy (DMD) is an X-linked recessive disorder, primarily affecting children around age four at an incidence of 1:5000 with an average life span of 30-40 years. DMD is due to a defect in the DMD gene encoding the dystrophin protein, which eventually leads to progressive muscle degeneration [1,2]. The dystrophin gene encodes typically for a functional dystrophin protein. Its primary role is to link the cytoskeleton with the extracellular matrix in cardiac and skeletal muscles via the dystrophin-glycoprotein complex (DPG).

Due to the defect in the DPG, the result is frail muscles, contraction-induced damage, necrosis, and inflammation. This eventually leads to the functional myofibers being taken over by fibrous and fatty connective tissue [3]. The most commonly affected muscle degeneration sites are the skeletal muscles, especially the lower limbs leading to calf pseudohypertrophy. The damaged muscle fibers become gradually replaced by fat and fibrotic tissue leading to progressive weakness and muscular atrophy. Other essential areas of disruptive myofiber integrity are the cardiac striated muscles and respiratory muscles, which leads to early death [4].

This disease is currently incurable; most teens end up having to use a wheelchair very early on. There are a few treatment options that help delay disease progression, including anti-inflammatory glucocorticoids, drugs to treat heart symptoms, physical therapy, and breathing assistance. Still, none have proven to increase the survival rate [5].

This study's objective is to investigate induced pluripotent stem cell (iPSC) therapy. It can be used to understand the pathogenesis of the disease, the positive benefits of new treatments, including genetic therapies, and any drawbacks that have occurred. Multiple adult cell populations with attributed muscle regenerative potential have been investigated for the past twenty years [5-9]. Human iPSCs are genetically engineered to an embryonic-like state that can potentially turn into almost any type of cell, which makes them ideal suitors for myogenic cells in DMD [10-18].

Methods

Literature was searched manually in PubMed with parallel strategies based on MeSH subheadings and regular keywords for data collection. The table below shows regular and MeSH keywords for the literature search (Table 1).

**Table 1:** Methods: Regular Keywords and MeSH Keywords using PubMed Database MeSH: medical subject headings

Regular Keywords	Database	Number of Results
Stem cell therapy	PubMed	195388
Muscular dystrophy	PubMed	35918
Becker muscular dystrophy	PubMed	12474
Duchenne muscular dystrophy	PubMed	12111
Dystrophin	PubMed	8061
Duchenne muscular dystrophy, gene	PubMed	4573
Muscular dystrophy, stem cell therapy	PubMed	1088
Stem cell, duchenne	PubMed	881
Duchenne muscular dystrophy, stem cell therapy	PubMed	583
Stem cell, duchenne, dystrophin gene	PubMed	347
Duchenne muscular dystrophy, satellite cells	PubMed	303
Duchenne muscular dystrophy, induced pluripotent stem cell	PubMed	103
MeSH KEYWORDS		
Muscular dystrophy	PubMed	87
Stem cell	PubMed	60
Duchenne muscular dystrophy	PubMed	2

Studies were selected after applying the following inclusion criteria.

1. Paper published in English

2. Papers within the past five years

3. The study types were a clinical trial, review articles

4. Only free full-text papers

This table shows the total number of articles after applying inclusion criteria in the following order (Table 2).

**Table 2:** Inclusion Criteria

Regular Keywords	Database	Number of Results
Stem cell therapy	PubMed	9,323
Muscular dystrophy	PubMed	677
Becker muscular dystrophy	PubMed	317
Duchenne muscular dystrophy	PubMed	311
Dystrophin	PubMed	138
Duchenne muscular dystrophy, gene	PubMed	133
Muscular dystrophy, stem cell therapy	PubMed	48
Stem cell, duchenne	PubMed	33
Duchenne muscular dystrophy, stem cell therapy	PubMed	21
Duchenne muscular dystrophy, satellite cells	PubMed	10
Duchenne muscular dystrophy, induced pluripotent stem cell	PubMed	15
Stem cell, duchenne, dystrophin gene	PubMed	8

## Review

Pathophysiology of DMD and current treatment options

The most critical aspect of the disease's progression is mutations of the DMD gene, which leads to a dysfunctional dystrophin protein. This protein plays a crucial role in providing membrane stability and anchoring the basal lamina to the cytoskeleton. A defective protein eventually leads to membrane instability, ensuing progressive muscle degeneration [19,20]. The most common muscular dystrophies are Becker's' muscular dystrophy and Duchene's muscular dystrophy; these diseases have over 1000 different genetic mutations, which can lead to different phenotypes causing a wide variety of responsiveness to therapy [20-22]. Currently, the condition is incurable, but DMD patients need support, especially when they become teens. Most teens transitioning into adult life are incredibly independent. DMD patients are usually wheelchair-bound by their teenage years, so they need a tremendous amount of support with healthcare, housing, transportation, education, employment, relationships with others, and activities of daily living [20-22]. There have been clinical trials of cell therapies in muscular dystrophies tested between 2012 and 2014. 

A stem cell is a primitive cell with the ability to reduce inflammation, fight apoptosis; self replicate and differentiate into multiple tissues (e.g., bone, cartilage, fat, muscle). Stem cells circulate throughout the body all through life. During stem cell retrieval, a local anesthetic is applied to the area where the sample is taken. The stem cell sample is then harvested and undergoes modification to suit the needs for a specific disease. Afterward, it is re-administered to the patient for therapeutic effects.

Stem cell therapies have been around for decades and have shown a favorable approach to treat certain neurodegenerative disorders, including DMD [23]. Human-induced pluripotent stem cells are adult cells that have been genetically modified to an embryonic state, which gives it the ability to turn into any type of cell essentially.

There are two ways to generate iPSCs: integrating vs. non-integrating. Examples would be retrovirus and proteins/ messenger ribonucleic adic (mRNA), respectively. However, retroviral integration is controversial due to the potential to activate specific oncogenes. mRNAs are known to reprogram cells at a higher efficiency also [24]. A significant limitation for in vitro studies and cell-based studies is collecting sufficient stem cells.Induced pluripotent stem cells (iPSCs) can eliminate this challenge. In previous studies, skeletal myogenic progenitors of embryonic stem cells (ESC) and iPSCs using the expression of pax 7. Pax 7 is part of the satellite cell niche; satellite cells are precursors to skeletal muscle cells [25-27]**.**

In DMD, the lack of dystrophin also causes an influx of calcium, which leads to destructive pathological changes. Exon skipping in DMD myotubes suppresses calcium influx compared to untreated DMD myocytes, which is an iPSC cell-based model done in vitro, a valuable tool to evaluate the pathogenesis of the disease [5]. Shoji et al. also reported a pronounced calcium influx only in DMD myotubes [28, 29].

After genetic modification, the stem cells expressed myogenin and myosin heavy chain (MHC), which are markers of muscle differentiation and a vital sign for a therapeutic advantage. Pax 7 also leads to increased dystrophin distribution and acetylcholine receptors, which indicate an improvement in neuromuscular ability. The expression of dystrophin also improves fibrosis [5, 28, 29]. The promises were especially apparent when mdx mice were injected, and the cells fused with older muscle fibers, which labeled the sample as eligible for muscle regeneration [30].

Another approach: genetic therapy compared to induced pluripotent stem cell therapy

There are two approaches to induce myogenic progenitors and precursor cells from iPSC:

1. Reprogramming with muscle-specific transcription factors (e.g., pax 7) is also known as a transgene approach, and

2. A step-wise induction of skeletal muscle cells utilizing small molecules or cytokines activates or inhibits specific pathways in the myogenesis process, also known as a transgene-free approach.

The transgene-free approach is safer but less efficient than the transgene approach.

While both show promising effects in disease modeling, drug screening/toxicity testing, cell-based therapy, and regenerative medicine, there is still a lot of room for improvement in human cells' effectiveness.

Multiple cell and gene-therapy studies have been conducted on satellite cells, the primary skeletal muscle stem cells involved in muscle regeneration [5]. For genetic corrections, they use CRISPR-Cas9 technology to delete exons 45-55 of DMD. This technology reframes the DMD transcript in human iPSC derived skeletal myotubes and cardiomyocytes, which lead to stable dystrophin protein and membrane stability [31]. A study was done in 2016, using the mdx mouse model, where they induced exon deletion and recovered dystrophin expression. This was achieved by the delivery of adeno-associated virus short palindromic repeats (CRISPR-Cas9) endonucleases paired with RNAs. Adeno-associated virus (AAV)-DMD-CRISPR treatment helped improve muscular function [32]. In 2019, a study was done on human patients using an oligonucleotide to induce exon 51 skipping in patients lacking exon 50. The report shows that dystrophin expression was allowed at 0.5% of the average level after one year of treatment [33].

Interpretation, analysis and knowledge gap

Based on previous findings, it is apparent that induced pluripotent stem cell therapy and genomic editing for treating Duchenne's muscular dystrophy has proven to improve muscular function drastically in mice. We compared the myogenic potential of iPS cells using transgene and transgene-free approaches. The transgene approach is more efficient. Pax7-induced iPS cells proliferate into myogenic progenitors and, when injected into mice, can lead to contractile improvement, muscle regeneration, and solidify our future for therapeutic approaches in muscular dystrophies [34].

Also, based on recent analysis and data, mesangioblasts and satellite cells show the most promising approach for functional recovery of treated muscles [11,34]. Embryonic cells, such as mesenchymal cells, including mesenchymal stem cell therapy, showed improvement in the vastus intermediate muscle on magnetic resonance imaging (MRI). A considerable limitation is that the study has been conducted mostly on mdx mice; we need to reproduce human muscle disease and monitor clinically relevant outcomes, which is achievable by creating an efficient in vitro skeletal muscle model [35]. A considerable knowledge gap is that we're not sure if a human counterpart can be identified and treated, due to the substantial differing aspects of species, specifically somatic cells [5]. Another approach we could attempt is a novel stem cell approach. Described as ex vivo fusion of healthy and dystrophin-deficient myoblast to create dystrophin expressing chimeric cells. When the mdx mouse model was tested, there was confirmed myogenic potential and proliferation up to 21 days.

## Conclusions

When we started this topic our main research questions towards DMD were regarding the pathophysiology, the genes and proteins responsible, the current treatment options, life expectancy, the evolution of treatment, and how stem cell therapy is adapting to the future of human clinical studies. 

In conclusion, DMD is, unfortunately, an X-linked recessive disorder affecting kids. Due to this disease's current incurable state, early wheelchair use is quite common. This ultimately results in cardiac failure and death by the third decade of life therefore, symptomatic and social support has to be provided. Despite all of these supportive measures, there has been no improvement in the survival rate. The treatment has evolved due to the promising effects of stem cell therapy, specifically induced pluripotent stem cell therapy, and genetic medicine. Stem cell therapy fits into the future of human clinical studies because it has been proven in mdx mice models prior, so we believe a breakthrough is imminent in the near future with human trials.

Our paper is important because it highlights the essential aspects of the disease, the patient's life span, and current treatments while mitigating the benefits of ongoing trials in stem cell therapy vs. genetic therapy in mdx mouse models. What we recommend to do moving forward is to discover if we can successfully perform the induced pluripotent stem cells in vitro on human specimens in healthy vs. disease muscle and compare results. Also, to explore if we can improve the lifespan of patients or prophylactically prevent the muscle damage from worsening by administering stem cells or genetic therapy once we have established a diagnosis.
